# Impact of Serial Casting on Autonomic Nervous System Responses during Virtual Reality Tasks in Children with Cerebral Palsy: A Pilot Study Comparing Orthoses and Barefoot Conditions

**DOI:** 10.3390/brainsci14101000

**Published:** 2024-09-30

**Authors:** Marisa de Paula Paro, Raísa Marques de Sousa, Juliana Perez Martinez, Amanda Orasmo Simcsik, Marina Junqueira Airoldi, Rodrigo Martins Dias, Íbis Ariana Peña de Moraes, Fernando Henrique Magalhães, Carlos Bandeira de Mello Monteiro, Talita Dias da Silva-Magalhães

**Affiliations:** 1 Graduate Program in Medicine (Cardiology), Escola Paulista de Medicina, Federal University of São Paulo (EPM/UNIFESP), São Paulo 03828-000, Brazil; 2 Graduate Program in Physical Activity Sciences, School of Arts, Science and Humanities of University of São Paulo (EACH-USP), São Paulo 03828-000, Brazil; 3 Therapies Centro de Reabilitação Intensiva, Campinas 13098-324, Brazil; 4 Graduate Program in Rehabilitation Sciences, Faculty of Medicine, University of São Paulo (FMUSP), São Paulo 01246-903, Brazil; 5 Department of Physiotherapy, Federal University of Juiz de Fora, Campus Governador Valadares, Governador Valadares 36036-900, Brazil; 6 Department of Physical Therapy, Faculty of Sciences and Technology (FCT/UNESP), State University of São Paulo, Presidente Prudente 14884-900, Brazil; 7 Graduate Program in Bioengineering, University Brazil, São Paulo 05508-010, Brazil

**Keywords:** cerebral palsy, plaster cast, autonomic nervous system, heart rate, virtual reality

## Abstract

Cerebral palsy (CP) is a group of movement disorders that impair posture and mobility, often leading to spasticity and joint contractures. Interventions like serial casting are commonly used to improve joint mobility and manage spasticity in children with CP. However, its effects on the autonomic nervous system (ANS) remain unclear. This study aimed to evaluate the effects of serial casting and ankle–foot orthoses (AFOs) on ANS responses during a virtual reality (VR) standing task, comparing these interventions with a barefoot condition. Thirty children with CP were randomized into three groups (*n* = 10 per group): serial casting, AFOs, and barefoot. Heart rate variability (HRV) was used to assess ANS responses across three phases: seated rest, VR task, and recovery. The results showed that the serial casting group exhibited higher sympathetic activity during rest compared to the other groups, but had a reduced sympathetic response during the VR task. Additionally, the serial casting group displayed a more pronounced parasympathetic rebound during recovery, similar to the orthoses and barefoot groups. While serial casting provides essential joint stability, it alters ANS response patterns, leading to heightened sympathetic activation at rest, without providing significant improvements in ANS behavior during physical activity.

## 1. Introduction

Cerebral palsy (CP) is a group of movement and posture disorders caused by injuries or non-progressive disturbances in the developing fetal or infant brain. These motor disorders are often accompanied by challenges in sensation, perception, cognition, communication, and behavior, as well as epilepsy and secondary musculoskeletal issues, leading to delays in neuropsychomotor development [1]. One of the most prominent symptoms of CP is spasticity, which increases muscle tension, restricts both passive and active joint mobility, and contributes to joint contractures. Spasticity impairs motor function, leading to discomfort, diminished self-esteem, and reduced quality of life. Additionally, it raises the risk of complications, such as infections, thrombosis, bedsores, irreversible contractures, and joint deformities, complicating both rehabilitation and self-care [2].

Individuals with CP often face not only motor impairments but also increased cardiovascular risks due to reduced mobility and sedentary behavior, which heighten the likelihood of chronic conditions like heart disease and hypertension [3]. Furthermore, children with CP are more prone to autonomic nervous system (ANS) dysfunction, which can further exacerbate cardiovascular problems [4,5,6]. Studies show that children with CP exhibit impaired heart rate variability (HRV), a key indicator of ANS function, particularly in response to postural changes [7]. Reduced HRV is associated with increased cardiovascular events and a higher risk of mortality in this population [8]. Therefore, early interventions that target both cardiovascular fitness and motor impairments are crucial for preserving long-term health and functional capacity [9].

To address both motor impairments and cardiovascular risks, rehabilitation programs for individuals with CP often focus on improving range of motion (ROM), functionality, and overall quality of life. Maintaining proper joint alignment and lower limb ROM is essential for stabilizing postural control, preserving musculoskeletal health, and reducing cardiovascular strain, as decreased mobility in CP can lead to a sedentary lifestyle, increasing the risks of hypertension and heart disease [3]. The progressive loss of ROM, particularly in the lower limbs, further compounds these risks by limiting physical activity, necessary for cardiovascular fitness [10].

Improved joint alignment can enhance ANS regulation by promoting better weight distribution and balance, leading to more efficient functional task performance, such as standing and walking. Proper alignment reduces the physical strain and effort required to maintain posture, which diminishes unnecessary sympathetic activation as this activation at rest leads to higher heart rate and blood pressure which are the principal causes of cardiovascular events during adulthood [9,11]. This decrease in physical exertion helps lower cardiovascular stress as the body no longer needs to compensate for imbalances or instability, allowing for more controlled, energy-efficient activity [8,9].

Thus, interventions through targeted therapies, such as ankle stabilization, play a critical role in slowing ROM decline, improving joint alignment, reducing spasticity, and enhancing mobility, helping to preserve function and reduce the strain associated with impaired movement patterns [3,4,6,11].

A well-established ankle stabilization intervention is the use of ankle–foot orthoses (AFO). Studies have demonstrated that consistent use of AFOs, especially for 7 h or more per day, is effective in maintaining or increasing ROM and enhancing mobility, which in turn reduces the cardiovascular stress associated with higher energy demands during functional tasks [12,13]. However, challenges in the use of AFOs arise as they can be easily removed, which often leads to inconsistent use, reducing effectiveness. Families frequently struggle with consistent monitoring and enforcing the use of AFOs to the level recommended by therapists, which undermines their ability to maintain joint alignment and prevent contractures [12,14].

Considering the difficulties with maintaining continuous use of AFOs, serial casting interventions have become increasingly utilized due to their ability to provide prolonged therapeutic effects. By applying a series of casts to gradually stretch muscles over several days, serial casting can effectively reduce contractures, increase joint flexibility, and improve mobility, particularly in the short term [15,16]. According to Novak et al. (2020) [17], serial casting presents high efficacy in reducing or eliminating early to moderate contractures, significantly improving joint alignment and postural control. This improvement leads to better functioning in tasks such as standing and walking, which are critical for children with CP [17,18,19]. However, potential adverse effects, such as skin irritation, pressure sores, and discomfort, may arise due to prolonged use. These complications are generally manageable with careful monitoring and adjustments, although they can occasionally impact daily functional tasks [19]. In addition, the immobilization and heaviness of the cast could limit physical activity, which may impact the cardiovascular system during the cast treatment [20,21].

While the musculoskeletal benefits of using serial cast are well-established, as far as we know, the impacts on the ANS and cardiovascular function remain unexplored. Given that children with CP often experience ANS dysfunction, which affects cardiovascular health, it is essential to assess whether the increased joint stability provided by serial casting could inadvertently overload the ANS during functional tasks like standing.

Thus, we organized a randomized clinical trial to analyze the effects of serial casting on autonomic nervous system responses in children with CP. The study compared three conditions: serial casting, orthoses, and barefoot (which offers no external foot support or stability). By examining these varying levels of joint stability, our aim was to assess how each condition influences ANS responses during functional tasks. To evaluate the autonomic function, HRV analysis was selected as a non-invasive measure, which can provide insights into the balance between sympathetic and parasympathetic activity. HRV is widely recognized as a sensitive marker for ANS regulation, making it an appropriate tool for assessing physiological responses to interventions like AFOs and serial casting.

To engage the participants and ensure the use of a functional task that required both motor and postural control, we utilized a non-immersive virtual reality (VR) standing task. VR-based interventions offer unique advantages in this context: they provide sensory feedback, encouraging physical activity and motivating children with CP. Moreover, research has shown that VR can stimulate moderate physical activity, enhancing both motor performance and cardiovascular function by promoting balanced energy expenditure and recovery processes [22,23,24]. This approach also aligns with previous studies indicating that VR can be effectively used to stimulate ANS responses in various conditions, making it a valuable tool for rehabilitation and analysis of HRV. Therefore, VR tasks enable the assessment of how joint stability interventions impact ANS regulation in children with CP.

In light of this approach and the study’s aims, we hypothesized that both serial casting and AFOs would enhance autonomic control more effectively than the barefoot condition due to the provision of external support, improved joint stability, and better posture, leading to less extortion when practicing the virtual task. However, because serial casting involves constant immobilization, it may also lead to higher sympathetic activity during the functional task compared to AFOs which offer the flexibility of intermittent use.

## 2. Materials and Methods

### 2.1. Study Design

The current study is a pilot study that is part of a larger randomized clinical trial (RCT) as previously published in the trial protocol [25]. This preliminary study, based on an initial sample, aims to assess the feasibility and provide early insights into the intervention before conducting a full RCT with a more robust sample size. This pilot study followed the CONSORT guidelines for reporting randomized clinical trials. The trial focused on the use of serial casting in the treatment of children with cerebral palsy; it was registered under RBR-5ynppq with the registration date of 20 June 2020.

The study received approval from the research ethics committee of the Faculty of Medicine of the University of São Paulo (CAAE: 30117820.5.0000.5505, approval date: 29 May 2023), and complied with Resolution 466/2012 of the National Health Council of 10/10/1996 which regulates research involving human beings and the Declaration of Helsinki (1964).

Following approval, all the participants who are minors signed an informed assent form, with additional consent provided by their legal guardians.

### 2.2. Participants

The study included 30 participants, all diagnosed with cerebral palsy, of both sexes aged between 5 and 12 years. The research was conducted at the Therapies Centro de Reabilitação Intensiva in Campinas, SP.

As inclusion criteria, we considered (1) children 3–12 years of age based on the definition of the Brazilian Society of Pediatrics [26]; (2) a diagnosis of cerebral palsy, GMFCS classification levels I, II, or III; and (3) the ability to understand simple commands in the virtual game, which was presented beforehand. Conventional physiotherapy sessions performed by each child were not considered, as participants were instructed to maintain their normal routine, with or without therapy.

Additionally, we considered as non-inclusion criteria (1) individuals who underwent bone surgery and/or arthrodesis; (2) applied botulinum toxin or serial plaster in the previous six months; (3) underwent musculotendinous surgery in the previous year; and (4) participants receiving intensive treatment during the application of the serial cast.

Exclusion criteria consisted of (1) participants who experienced pain from the serial cast; (2) had an allergy to the material; (3) faced medical complications; and (4) abandoned the protocol.

### 2.3. Interventions

After providing informed consent, the legal guardians completed a form to provide key demographic and clinical information, including the child’s age, height, weight, sex, use of walking aids, and use of orthoses. These data were collected to accurately characterize the participants.

To classify the children and adolescents with CP, the gross motor function classification system (GMFCS) was used. This system categorizes individuals based on their spontaneous movement, with a particular focus on sitting, transfers, and mobility. The GMFCS divides participants into five levels, according to their functional limitations, mobility, and movement quality. For the current study, only participants classified as GMFCS levels I, II, and III were included.

All participants then underwent a motor function assessment using the gross motor function measure (GMFM), focusing specifically on Dimensions D and E. These dimensions assess the child’s ability to move or perform specific motor tasks, with each task scored on a scale from zero (inability to perform) to three (full, independent performance). Dimension D measures standing abilities, while Dimension E evaluates walking, running, and jumping. These dimensions were selected because they most effectively assess the functional standing and locomotor skills relevant to the study’s objectives.

After the GMFM assessment, participants in the serial casting group received the cast application, which is described in detail in the following section.

#### 2.3.1. Cast Application

Range of motion in the joints was measured using Resistance-1 (R1) and Resistance-2 (R2), where R1 represents the first point of resistance during passive dorsiflexion of the ankle, performed with the joint in alignment, and R2 is the maximum range of motion that can be achieved passively [15]. These measurements were used in the current study to determine the ankle joint’s range of motion at the beginning of the serial casting protocol.

**Figure 1:**
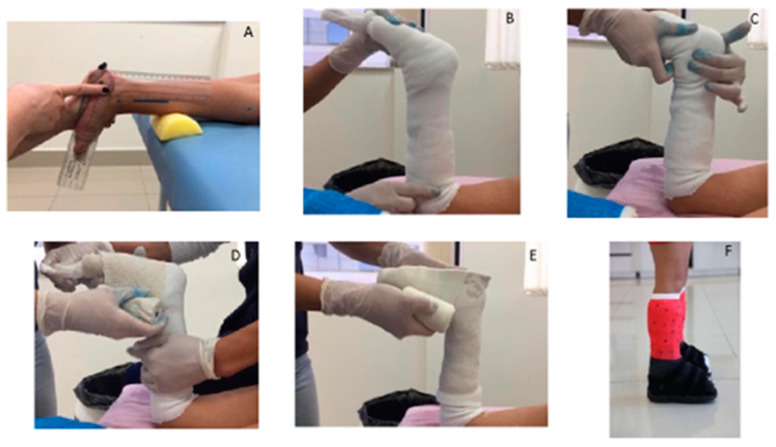
Procedures for cast application. (**A**): Range of motion measurement “R1”; (**B**): Applying the protective layer; (**C**): Positioning on R1; (**D**): First layer of cast (powder cast); (**E**): Second layer of the cast (synthetic plaster); and (**F**): Support base.

The assessment was carried out under normal conditions, without orthoses or any assistive technology. After the evaluation, a custom-made cast was created for each child, according to the following steps: (1) Range of motion measurement: The child was positioned prone on a stretcher, with one lower limb in 90-degree knee flexion and the foot in a neutral position, aligned with the R1 measurement (Figure 1A). (2) Applying the Protective Layer: A foam padding (0.5 cm thick, density between 18 and 23) was applied to protect bony prominences, with custom cutouts made to fit each child and adding the tubular mesh which was placed over the entire area that would receive the cast to ensure even coverage (Figure 1B). (3) Positioning on R1: Performed to start the casting (Figure 1C). (4) First layer of cast: A powder cast was applied to the foot, extending up to the calf. During this step, the contours of the child’s foot were carefully marked (Figure 1D). (5) Second layer of Cast: A second layer made of fiberglass (synthetic material) was applied to strengthen the cast. This layer extended from the toes to just below the popliteal fossa (Figure 1E). (6) Support base: Once the cast dried, necessary adjustments were made with shims while the child was seated, and a final verification was performed in the standing position with a specific shoe for casting to ensure optimal stability and support (Figure 1F).

**Figure 2:**
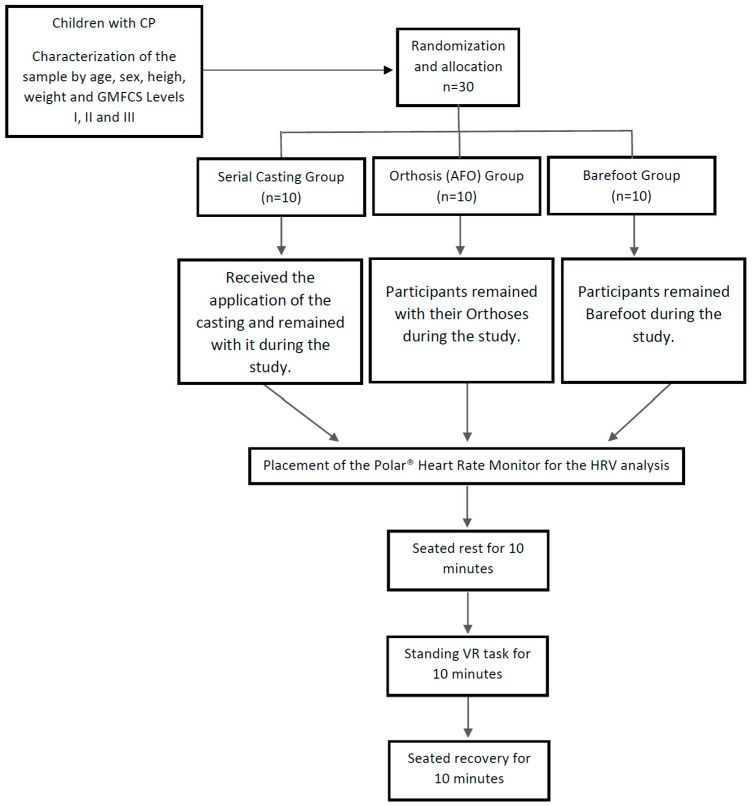
Setting of the study. CP: cerebral palsy; GMFCS: gross motor function classification system; AFO: ankle–foot orthoses; HRV: heart rate variability.

After the cast application in the serial casting group, all participants including those in the orthoses and barefoot groups were prepared for HRV data collection to assess ANS responses under different conditions. HRV was measured in three distinct phases: first, the participants were seated for ten minutes to capture the ANS response during rest, followed by a ten-minute standing virtual task to observe how joint stability influenced ANS function during activity. Finally, participants returned to a seated position for ten minutes to assess ANS recovery after the functional task. This protocol allowed for comprehensive evaluation of ANS regulation across different states: rest, active standing, and recovery (Figure 2).

#### 2.3.2. Virtual Reality Task—MoveHero

**Figure 3:**
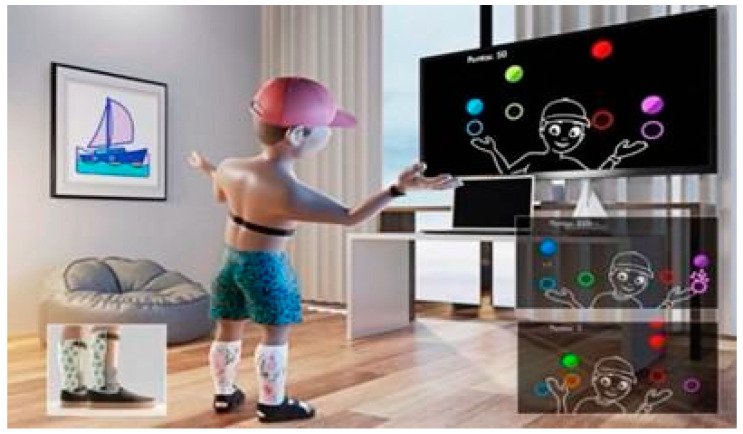
Child wearing a serial cast playing the virtual game “MoveHero” while wearing a polar belt to collect heart rate variability data.

The virtual game “MoveHero” was developed at the School of Arts, Sciences, and Humanities of the University of São Paulo and used in several studies with CP [27], down syndrome [28], and autism spectrum disorder [29,30] to measure performance and provide physical activity. In this game, spheres fall in four imaginary columns on the computer screen to the rhythm of a song selected by the researcher for 5 min. The task is to prevent the spheres from reaching the bottom of the screen. The spheres can only be stopped when they reach four circles placed in parallel (at two different heights), two to the left and two to the right of the participant, named targets 1, 2, 3, and 4, from left to right. The game captures the participant’s movements via a webcam, requiring no physical contact. The participant moves their upper limbs at a distance of 0.5 to 1 m from the computer screen. The participant needs to wait for the spheres to overlap one of the target circles (at which point the circle changes color to green). The game thus requires the participant to anticipate the movement to reach the spheres within these circles (Figure 3). Feedback on successful hits is provided through a number (+1) that appears next to the sphere when it is hit within the target. Additionally, the total score is displayed in the upper left corner of the screen, with 10 points awarded for each successful hit.

### 2.4. Outcomes

#### 2.4.1. Heart Rate Variability Analysis

HRV is a simple, reliable, inexpensive, and non-invasive measure used to capture autonomic impulses. Its widespread use, cost-effectiveness, and ease of data acquisition make HRV an appropriate choice for interpreting cardiac autonomic function and a promising clinical tool for assessing and identifying physiological alterations. Fluctuations in HRV patterns provide early and sensitive diagnoses of physiological behavior and health status [6,31]. In the current study, HRV was evaluated as a control variable during rest, the practice of “MoveHero”, and recovery.

All heartbeat records were collected using a Polar^®^ RS800CX heart rate monitor. HRV was recorded while the participant was seated for 10 min at rest, for 10 min while standing during the “MoveHero” task, and for 10 min during recovery.

For HRV data analysis, 256 consecutive RR intervals were used, followed by manual filtering. Kubios HRV software v. 3.3.1 was used for analysis.

#### 2.4.2. Subsubsection Poincaré Analysis

A Poincaré plot was used to translate the RR intervals into geometric patterns, permitting HRV analysis through the geometric or graphic properties of the resulting patterns. SD1 represents the short-term variability of continuous RR intervals, and SD2 represents the long-term variability from which the SD1/SD2 ratio can be calculated. Poincaré plot analysis is based on nonlinear dynamics [23].

#### 2.4.3. Detrended Fluctuation Analysis (DFA)

DFA was adopted to calculate the fluctuation in the root mean square of the integral and purify the time series, allowing the detection of intrinsic auto similarity embedded in non-stationary time series. The DFA graph is not strictly linear but consists of two distinct regions with different curves, separated by one point, indicating a short-term fractal scale exponent (α1) during periods of 4–11 beats (or 4–13) and a long-term exponent (α2) for longer periods (>11 beats) [32].

#### 2.4.4. PNS, SNS, STRESSI

The sympathetic index (SNSi) and parasympathetic index (PNSi) were proposed to separately assess the synergistic functions of the autonomic nervous system by analyzing the timing of heartbeats based on the observation that cholinergic and adrenergic impulses have different temporal dynamics. Values of PNSi and SNSi around zero indicate that the respective activity parameters (parasympathetic and sympathetic) are, on average, equal to the normal population mean. The Baevsky stress index (Stressi) is a geometric measure of HRV that reflects cardiovascular system stress, with high Stressi values indicating reduced variability and high sympathetic cardiac activation [33,34,35].

### 2.5. Sample Size, Randomization and Allocation

The sample size was calculated using GPower 3.1.5 statistical software based on the main outcome measures (i.e., HRV). Considering a power of 0.80, an alpha of 0.05, and an effect size of 0.65 (Cohen’s d), the estimated sample size required was 30 participants, with 10 in each group.

The participants were randomly assigned to one of three groups, each comprising 10 individuals: one group using serial Casting, one group using Orthoses (AFOs), and one group who were Barefoot. Randomization was performed in a 1:1:1 ratio and repeated as necessary to ensure homogeneity across groups, avoiding bias, with each group having a similar distribution of participants by age and GMFCS classification. Blinding was not possible for participants or researchers due to the nature of the interventions.

### 2.6. Statistical Analysis

The sample characterization data are presented as means and standard deviations (SD) or as absolute and relative values. Numerical data were analyzed using Multiple Analysis of Variance (MANOVA), and categorical data were analyzed using the chi-square test. For the HRV indices, MANOVA was used, considering 3 factors (groups: Casting, Orthoses, Barefoot) and 3 moments (Rest, Activity, Recovery), with repeated measures for the last factor. The LSD (Least Significant Difference) post hoc test was applied. Partial Eta squared (ηp^2^) was reported to measure the effect size, interpreted as small (effect size > 0.01), medium (effect size > 0.06), or large (effect size > 0.14) [36]. Graph data are presented as mean and standard error. The statistical package used was SPSS version 26.0, and *p* values < 0.05 were considered significant.

## 3. Results

In total, 30 children were evaluated. The demographic data and comparisons between the three evaluated groups are shown in Table 1, demonstrating that the groups were homogeneous with respect to the variables presented in the table. This homogeneity minimizes bias in the results across the different support bases.

### 3.1. Heart Rate Variability

The MANOVA revealed a significant main effect for the Moments factor (F_16,12_ = 5.53; *p* = 0.002, ηp^2^ = 0.88; Wilks’ λ = 0.119), with no significant interactions between the factors. Separate ANOVAs for the DFA, Poincaré, PNSi, SNSi, and Stressi indices are detailed in the following sections.

### 3.2. DFA and Poincaré Indices

**Figure 4:**
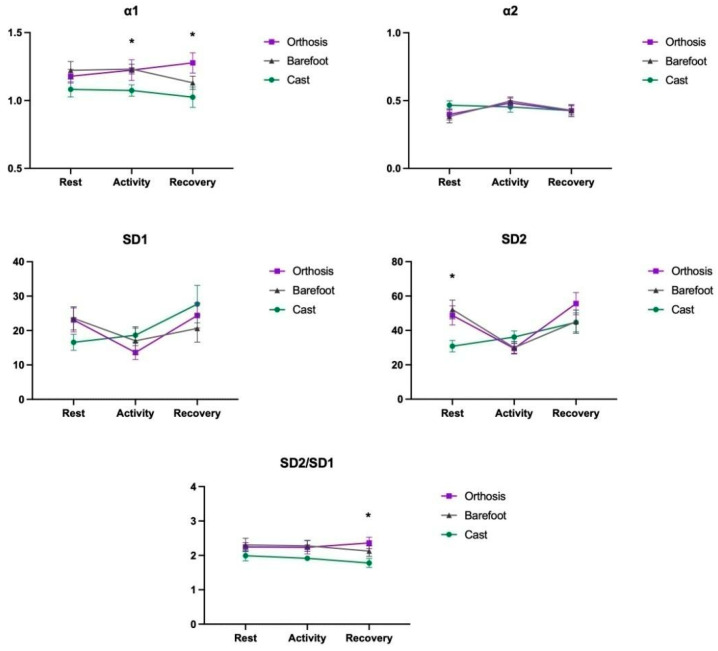
Representation of the mean and standard error of the DFA and Poincaré indices between groups (Casting, Orthoses, and Barefoot) and moments (Rest, Activity, and Recovery). * means *p* < 0.05.

A main effect was identified for the Moments factor in the α2 index (F_2,54_ = 4.41; *p* = 0.017, ηp^2^ = 0.14), SD1 index (F_2,54_ = 8.13; *p* = 0.002, ηp^2^ = 0.23), and SD2 index (F_2,54_ = 18.77; *p* < 0.001, ηp^2^ = 0.41). Additionally, a main effect for the Group factor was found in the α1 index (F_2,27_ = 3.44; *p* = 0.047, ηp^2^ = 0.20), and there was an interaction between Groups and Moments for the SD1 (F_4,54_ = 2.97; *p* = 0.036, ηp^2^ = 0.18) and SD2 indices (F_4,54_ = 5.96; *p* = 0.001, ηp^2^ = 0.30). These results suggest that the Casting group had an α1 value closer to 1 (M = 1.06) compared to the Barefoot (M = 1.19; *p* = 0.050) and Orthoses (M = 1.22; *p* = 0.020) groups. Furthermore, during the Activity moment, the groups showed an increase in α2 (M = 0.47) and a decrease compared to the other moments (Rest M = 0.41, *p* = 0.013; Recovery M = 0.42, *p* = 0.024), except for the Casting group that did not demonstrate this increase in α2. SD1 and SD2 values were higher during the activity (M = 16.4; M = 31.8, respectively) compared to the other moments (Rest M = 21.0, *p* = 0.009 and M = 43.9, *p* < 0.001; Recovery M = 24.2, *p* = 0.002 and M = 48.5, *p* < 0.001), except for the Casting group that did not show this significant difference. Post hoc comparisons demonstrated that, for the SD2 index at rest, the Casting group (M = 16.6 bpm) had a lower value than both the Orthoses (M = 23.1 bpm; *p* = 0.015) and Barefoot groups (M = 23.5 bpm; *p* = 0.005). However, this difference was not observed during the other assessment moments. Finally, in the SD2/SD1 index, the Casting group presented a lower value (M = 1.7) than the Orthoses group (M = 2.4, *p* = 0.011) during recovery (Figure 4) (for associated data, please see Supplementary Materials, Table S1).

### 3.3. PNSi, SNSi, and Stressi

**Figure 5:**
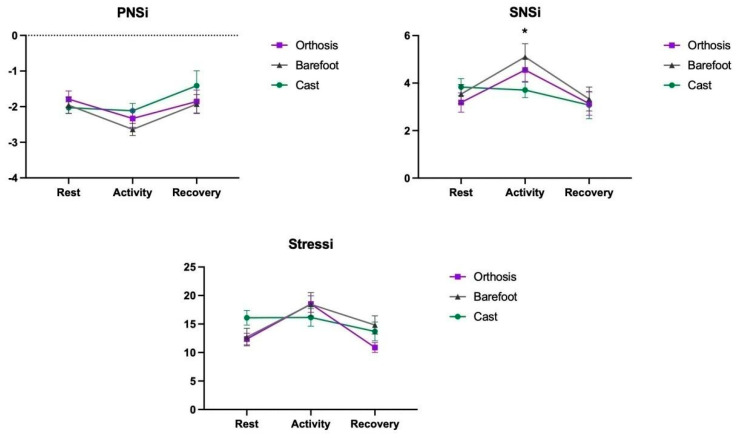
Representation of the mean and standard error of the PNSi, SNSi and Stressi indices between groups (Casting, Orthoses, and Barefoot) and moments (Rest, Activity, and Recovery). * means *p* < 0.05.

A main effect was found for the Moments factor in the PNSi (F_2,54_ = 11.48; *p* < 0.001, ηp^2^ = 0.29), SNSi (F_2,54_ = 19.22; *p* < 0.001, ηp^2^ = 0.41), and Stressi (F_2,54_ = 20.82; *p* < 0.001, ηp^2^ = 0.43) indices, with no significant main effect for the Group. Additionally, an interaction was found between the Moment and Group factors for the SNSi (F_4,54_ = 3.27; *p* = 0.024, ηp^2^ = 0.19) and Stressi indices (F_4,54_ = 4.59; *p* = 0.003, ηp^2^ = 0.25). These results indicate that during the Activity moment, the groups had lower PNSi (M = −2.36) compared to the other moments (Rest M = −1.92, *p* = 0.013; Recovery M = −1.73, *p* = 0.024), except for the Casting group that did not show this significantly lower value. For the SNSi and Stressi indices, there was an increase during the activity (M = 4.45; M = 17.7, respectively) compared to the other moments (Rest M = 3.51, *p* = 0.001 and M = 13.72, *p* < 0.001; Recovery M = 3.18, *p* < 0.001 and M = 13.13, *p* < 0.001), except for the Casting group that did not show this significant increase. Post hoc comparisons demonstrated that the SNSi index during the Activity moment in the Casting group (M = 3.70 bpm) was lower than in the Barefoot group (M = 5.10; *p* = 0.042); this difference was not observed in the other assessment moments. Lastly, in the Stressi index, only the Casting group showed no change from the Rest moment to the Activity moment (M = 16.09 and M = 16.16, *p* = 0.961) (Figure 5) (for associated data, please see Supplementary Materials, Table S1).

## 4. Discussion

This preliminary study investigated changes in cardiac autonomic modulation through heart rate variability analysis during a standing activity in a virtual reality environment, comparing the effects of serial casting, AFOs, and barefoot conditions in individuals with cerebral palsy (CP). Our hypothesis was partially confirmed, as the group using orthosis demonstrated better ANS responses. However, these responses were unexpectedly similar to those observed in the Barefoot group, suggesting that while orthoses provided external stability, they did not lead to a significant advantage in ANS regulation over the barefoot condition. On the other hand, the Casting group demonstrated higher sympathetic activity at rest and did not exhibit increased demand during the VR activity compared to the other groups. Interestingly, this group also showed superior recovery post-activity, characterized by an increase in parasympathetic activity similar to that of the other groups.

The present findings suggest that while serial casting offers improved stability, the reduced variability in ANS response may indicate a compensatory mechanism when transitioning from rest to activity as compared to the orthoses and barefoot groups. However, due to the preliminary nature of this pilot study, caution is needed in interpreting these findings, and further research with a larger sample size is required. As described above, despite higher sympathetic activity at rest in the casting group, the individuals did not exhibit a corresponding increase in sympathetic activity during the VR activity. However, the casting group demonstrated more favorable post-exercise recovery, marked by an increase in parasympathetic activity. This suggests that while the autonomic responses during activity in the casting group differed from those in the other groups, the ultimate recovery response appears to be similar across all groups. This aspect is further explored below.

### 4.1. Impact of Serial Casting on Physical Activity

Serial casting, which involves applying multiple layers of plaster or fiberglass to an affected limb [19], significantly increases the weight and rigidity of the cast. This added weight can make movement more challenging, potentially leading to a reduction in overall physical activity levels. As noted in studies by Singer et al. (2001) [20] and Solanki et al. (2010) [21], the restrictive nature of serial casting, while beneficial for correcting deformities and improving joint mobility, inevitably limits the range of motion, often resulting in decreased daily physical activities during the treatment phase.

The higher sympathetic modulation at rest observed in our study might be attributed to the heavy and rigid nature of the cast, which not only restricts movement but also contributes to the discomfort experienced by the individual, particularly in children. Omololu et al. (2002) [37] noted that such physical limitations, caused by serial casting, are perceived by the body as chronic stressors, known to trigger an increase in sympathetic nervous system activity. This heightened sympathetic activation can persist even during rest, as the body remains in a state of mild stress due to immobilization and discomfort. Research by Schneider et al. (2023) [38] supports this, demonstrating that sustained physical stress significantly impacts the autonomic nervous system by increasing sympathetic activation, even at rest, as observed in our results.

When comparing serial casting with orthotic use or barefoot conditions, it is evident that less restrictive interventions allow for greater physical activity. Orthoses are designed to support and correct anatomical structures, without heavily compromising mobility, which may be associated with lower levels of sympathetic activity at rest and the expected increase during activity observed in individuals using orthoses. This adaptation of ANS to the activity did not occur in the children with the cast, suggesting that the reduction in physical activity associated with serial casting could lead to diminished HRV during activities.

Recent research by Maggio et al. (2017) [39] provides empirical evidence supporting this concept. The researchers observed a noticeable decrease in activity-related energy expenditure in adolescents with lower limb fractures who were immobilized with casts. These results highlight how immobilization reduces the metabolic demand typically associated with movement and weight-bearing, effectively lowering energy costs due to low physical activity levels.

Although no autonomic adaptation from rest to activity was observed in this preliminary study, there was a significant response from activity to recovery in the casting group: post-exercise, this group displayed a notable increase in parasympathetic activity, which is critical for promoting relaxation and physiological recovery after exertion. This parasympathetic surge suggests an effective recovery process, during which the body transitions from a state of stress and heightened alertness (caused by the VR activity and wearing the cast) to one of rest and recuperation. The increase in parasympathetic activity is beneficial as it helps lower heart rate, reduces blood pressure, and decreases muscle tension, thereby facilitating faster recovery and improving overall cardiovascular health. This is a particularly interesting result for long-term therapy, as it suggests that while the cast may restrict movement and initially increase sympathetic activity at rest, it does not exacerbate stress responses during physical activity, ultimately supporting an efficient recovery phase similar to that observed in the orthoses and barefoot groups.

### 4.2. Impact of Ankle and Calf Casts on Venous Return and Blood Flow

While casts are essential for improving range of motion and stabilizing and healing ankle and calf injuries, their restrictive nature can present significant physiological challenges, including reduced venous return, which leads to increased sympathetic activity. When a cast is applied to the ankle and extends to the calf, it exerts mechanical compression on the underlying tissues, including blood vessels. This compression can significantly impede venous return by restricting the natural movement of the calf muscles which play a critical role in facilitating blood flow back to the heart. The phenomenon of venous stasis, as a result of this restriction, is a well-documented response to reduced venous blood flow and can lead to discomfort and swelling in the affected limb [40,41,42]. This effect is particularly pronounced in immobilization, where the absence of muscle contractions fails to activate the muscle pump necessary for enhancing venous return.

Mansur et al. (2019) [40] describe how venous distension, due to reduced blood flow, triggers the activation of the sympathetic nervous system. This response is a physiological adaptation to maintain vascular tone and blood pressure in the face of diminished venous return. Sympathetic activation can lead to an increase in heart rate and vasoconstriction in other regions, as the body attempts to compensate for the reduced efficiency of blood flow caused by the cast.

Similarly, Lerebourg et al. (2020) [41] in their systematic review highlight that immobilization decreases flow velocities due to the inability to mechanically stress foot veins which is typically achieved through natural foot movements. The review points out that without the dynamic actions that stimulate the muscle pumps in the legs, venous return is compromised, leading to slower blood flow velocity and an increased risk of venous stasis.

The kinetic and kinematic alterations caused by immobilization contrast sharply with the effects of active movement. Active muscle contractions, particularly in the lower legs, significantly enhance blood flow velocities by activating the muscle pump mechanism, which is crucial for venous return. Craik et al. (2015) [42] emphasize that the frequency of these contractions plays a vital role in the kinetics of venous return, influenced by the type, intensity, speed, and duration of the activity. This understanding underscores the importance of considering therapeutic exercises and movements within the constraints of a cast in order to mitigate the adverse effects of immobilization.

### 4.3. Influence of Virtual Reality Therapy on ANS Response

The VR activity used in the present study successfully stimulated a level of physical activity sufficient to increase sympathetic modulation in children with CP in both the orthoses and barefoot groups. Notably, the activity also maintained an elevated sympathetic tone in children in the casting group. This observation suggests that VR activity may trigger an adaptation mechanism, where an initially high sympathetic tone primes the cardiovascular system to manage physical activity without necessitating additional stress responses. This finding aligns with research by Maggio et al. (2017) [39], which indicated that even minor reductions in physical activity due to immobilization could result in slight positive energy balances. Therefore, it is crucial to promote feasible physical activities during periods of immobilization to effectively manage energy balance.

Moreover, all groups exhibited significant parasympathetic recovery post-activity, which is a key finding of this pilot study. This outcome suggests that when the activity ceases, the system can quickly revert to a state of relaxation. This shift is facilitated by an already primed parasympathetic system, which is prepared to take over and drive the recovery process. Engaging in light physical activities such as those enabled by VR (which are safe even within the constraints of immobilization) could help mitigate reductions in metabolic rate and maintain cardiovascular health. These activities offer a valuable strategy to balance energy expenditure and recovery, essential for the overall well-being of children with CP.

### 4.4. Limitations and Future Research

The current study presents several limitations that should be mentioned. (1) As a pilot study, a relatively small sample size (with only 30 participants divided into three groups) may limit the statistical power and generalizability of the findings. (2) The influence of age on HRV: although Gasior et al. (2018) [43] provide normative HRV values for school-aged children, the impact of HRV during gameplay and the use of orthoses could still affect the results. This influence should be further analyzed in future studies. (3) The intervention was conducted over a short term, potentially overlooking the longer-term effects of serial casting, orthoses, and barefoot conditions on cardiac autonomic modulation in children with cerebral palsy. This short duration may not capture the full implications of these interventions on autonomic nervous system responses or the potential for adaptive changes over time.

Future studies on the effects of serial casting on autonomic function and cardiovascular health are essential due to the limited understanding of how immobilization influences these systems over time. Key areas for future studies include longitudinal assessments of heart rate variability and autonomic responses throughout and after the period of immobilization, the integration of rehabilitation protocols to mitigate negative impacts, and investigations into the psychological and behavioral effects of reduced mobility and increased discomfort. Addressing these areas will enhance our understanding of both the physiological and psychological dimensions of cast immobilization, ultimately improving treatment outcomes.

## 5. Conclusions

While serial casting provides crucial joint stability, it probably alters autonomic regulation, which may result in increased sympathetic activation at rest without a corresponding increase during the VR task. These findings suggest the importance of balancing the therapeutic benefits of joint stability with potential disruptions to autonomic function when developing rehabilitation strategies for children with cerebral palsy. Careful consideration is needed to optimize interventions that support both motor function and autonomic health, ensuring comprehensive and effective rehabilitation outcomes.

## Figures and Tables

**Table 1:** Characterization of the sample and results of the comparisons between Casting, Orthoses, and Barefoot groups.

Variable	Casting	Orthoses	Barefoot	*p*-Value
	Mean ± SD	Mean ± SD	Mean ± SD	
Age (years)	6.4 ± 1.6	8.5 ± 2.5	7.8 ± 2.1	0.141
Weight (kg)	21.2 ± 6.9	28.4 ± 9.7	28.7 ± 8.3	0.158
Height (meters)	1.15 ± 0.09	1.30 ± 0.17	1.24 ± 0.10	0.106
GMFM D	57 ± 30	49 ± 31	59 ± 35	0.956
GMFM E	41 ± 29	34 ± 30	42 ± 31	0.942
	*n* (%)	*n* (%)	*n* (%)	
Sex				
Female	6 (46)	3 (23)	4 (31)	0.387
Male	4 (24)	7 (41)	6 (35)	
GMFCS				
I	3 (50)	1 (17)	2 (33)	0.688
II	1 (14)	3 (43)	3 (43)	
III	6 (35)	6 (35)	5 (30)	
Gait device				
None	4 (25)	5 (36)	7 (44)	0.392
Walker	6 (43)	5 (36)	3 (21)	
Orthoses				
None	1 (25)	0 (0)	3 (75)	0.283
Shoes	2 (40)	1 (20)	2 (40)	
Orthoses	7 (33)	9 (43)	5 (24)	

Abbreviations: SD: Standard deviation; GMFM: Gross motor function measure; D: Dimension D of GMFM; E: Dimension E of GMFM; GMFCS: Gross motor functional classification system; I: level I; II: level II; III: level III.

## Data Availability

The raw data supporting the conclusions of this article will be made available by the authors on request.

## Supplementary Materials

The following supporting information can be downloaded at: https://www.mdpi.com/article/10.3390/brainsci14101000/s1. Table S1. Detailed results of Heart Rate Variability (HRV) variables.
