# Supplementary material for: Impact of Serial Casting on Autonomic Nervous System Responses during Virtual Reality Tasks in Children with Cerebral Palsy: A Pilot Study Comparing Orthoses and Barefoot Conditions
Source: Brain Sci. 2024 Sep 30;14(10):1000. doi: 10.3390/brainsci14101000 (PMC11506228; doi:10.3390/brainsci14101000)
Supplement: Supplementary file 1 [file brainsci-14-01000-s001.zip › brainsci-3186784-supplementary Table S1.pdf]

Supplementary Material:
Table S1. Detailed results of Heart Rate Variability (HRV) variables

Variable	Group	Moments	Mean	Standard Error	Confidence Interval 95%	
					Lower bound	Upper bound
**PNSi**	Cast	Rest	-2,029	,210	-2,460	-1,597
		Activity VR	-2,114	,209	-2,543	-1,685
		Recovery	-1,412	,342	-2,114	-,710
	Barefoot	Rest	-1,958	,210	-2,389	-1,526
		Activity VR	-2,639	,209	-3,068	-2,210
		Recovery	-1,927	,342	-2,629	-1,226
	Orthosis	Rest	-1,789	,210	-2,220	-1,358
		Activity VR	-2,333	,209	-2,762	-1,904
		Recovery	-1,856	,342	-2,558	-1,154
**SNSi**	Cast	Rest	3,833	,388	3,036	4,630
		Activity VR	3,708	,463	2,757	4,659
		Recovery	3,074	,523	2,002	4,146
	Barefoot	Rest	3,536	,388	2,739	4,333
		Activity VR	5,104	,463	4,153	6,055
		Recovery	3,330	,523	2,258	4,402
	Orthosis	Rest	3,183	,388	2,386	3,980
		Activity VR	4,553	,463	3,602	5,504
		Recovery	3,139	,523	2,067	4,211
**Stressi**	Cast	Rest	16,090	1,302	13,418	18,762
		Activity VR	16,160	1,708	12,656	19,664
		Recovery	13,700	1,416	10,794	16,606
	Barefoot	Rest	12,710	1,302	10,038	15,382
		Activity VR	18,450	1,708	14,946	21,954
		Recovery	14,830	1,416	11,924	17,736
	Orthosis	Rest	12,360	1,302	9,688	15,032
		Activity VR	18,505	1,708	15,001	22,009
		Recovery	10,885	1,416	7,979	13,791
**SD1**	Cast	Rest	16,590	3,102	10,226	22,954
		Activity VR	18,645	2,869	12,759	24,531
		Recovery	27,685	4,413	18,630	36,740
	Barefoot	Rest	23,545	3,102	17,181	29,909
		Activity VR	17,010	2,869	11,124	22,896
		Recovery	20,625	4,413	11,570	29,680
	Orthosis	Rest	23,090	3,102	16,726	29,454

		Activity VR	13,605	2,869	7,719	19,491
		Recovery	24,370	4,413	15,315	33,425
**SD2**	Cast	Rest	30,810	4,895	20,767	40,853
		Activity VR	36,160	3,367	29,252	43,068
		Recovery	44,720	6,257	31,882	57,558
	Barefoot	Rest	52,160	4,895	42,117	62,203
		Activity VR	29,940	3,367	23,032	36,848
		Recovery	45,110	6,257	32,272	57,948
	Orthosis	Rest	48,740	4,895	38,697	58,783
		Activity VR	29,525	3,367	22,617	36,433
		Recovery	55,630	6,257	42,792	68,468
**SD2/ SD1**	Cast	Rest	1,991	,159	1,665	2,318
		Activity VR	1,916	,150	1,609	2,223
		Recovery	1,780	,151	1,470	2,089
	Barefoot	Rest	2,304	,159	1,977	2,631
		Activity VR	2,279	,150	1,972	2,586
		Recovery	2,124	,151	1,814	2,433
	Orthosis	Rest	2,245	,159	1,918	2,571
		Activity VR	2,236	,150	1,930	2,543
		Recovery	2,363	,151	2,053	2,672
**α1**	Cast	Rest	1,082	,057	,965	1,199
		Activity VR	1,074	,054	,962	1,185
		Recovery	1,024	,067	,887	1,162
	Barefoot	Rest	1,222	,057	1,105	1,339
		Activity VR	1,231	,054	1,120	1,343
		Recovery	1,130	,067	,993	1,268
	Orthosis	Rest	1,179	,057	1,061	1,296
		Activity VR	1,224	,054	1,112	1,335
		Recovery	1,277	,067	1,139	1,414
**α2**	Cast	Rest	,466	,041	,381	,551
		Activity VR	,454	,035	,382	,526
		Recovery	,426	,040	,344	,509
	Barefoot	Rest	,383	,041	,299	,468
		Activity VR	,498	,035	,425	,570
		Recovery	,429	,040	,347	,512
	Orthosis	Rest	,399	,041	,314	,484
		Activity VR	,483	,035	,411	,556
		Recovery	,426	,040	,343	,508
